# Supplementary material for: Learning how a tree branches out: A statistical modeling approach
Source: PLoS One. 2022 Sep 21;17(9):e0274168. doi: 10.1371/journal.pone.0274168 (PMC9491565; doi:10.1371/journal.pone.0274168)
Supplement: S1 Appendix — (PDF) [file pone.0274168.s002.pdf]

Appendix A Algorithms for model fitting

This section gives the detailed structure of the two algorithms used for model selection. This first one selects the best explanatory variable according to the AIC criterion among a pool of candidates. The second one performs stepwise variable selection based on the AIC.

Algorithm 1: Algorithm for Model Fitting

```
// A function which finds the best explanatory variable relative to a baseline
   model using the AIC criterion.
Function Best_Explanatory_Variable(vars, data, baseline):
    Input: vars = (x_1, ..., x_k)          // A vector of the names of candidate variables.
    Input: data      // A matrix with data for the model fitting. The column headers are the variable names.
    Input: baseline                         // Model formula of the baseline model.
    Output: M_best  // The best model, according to the AIC criterion, obtained by adding one variable in
                        var to the baseline.
    Initialize C_M = ∅                      // C_M is the set of candidate models.
    k = |vars|
    for j = 1 to k do
        New_model_j := Fit(baseline + x_j)  // model formula is baseline model with one variable
                                               from vars
        C_M = C_M ⋃ New_model_j             // The set of candidate models gets larger.
    end
    Evaluate AIC for each model in C_M.
    V_Best := Variable from vars which gives a model with the lowest AIC.
    M_best := Model with the lowest AIC
    return M_best
    return V_Best
    return AIC(M_best)
End Function
```

// A function which performs stepwise selection on a set of candidate variables.

Function `Stepwise_Fit`($vars$, $data$, $baseline$):

Input: $vars = (x_1, \dots, x_k)$ // A vector of the names of candidate variables

Input: $data$ // A matrix with data for the model fitting. The column headers are the variable names.

Input: $baseline$ // Model formula of the baseline model.

Output: $\mathcal{M}_{best}$ // The best model consisting of the baseline and all possible variable from var based on AIC criteria

Declare $\mathcal{C}_{\mathcal{V}} = \{vars\}$ // The set of candidate variables

Initialize $\mathcal{M}_{best} = baseline$ // The current best model

Initialize $\mathcal{M}_{candidate}$ = NULL // A candidate model

Initialize $AIC_{Current} = AIC(baseline)$ // The AIC of the current best model

Initialize $AIC_{Previous} = AIC(baseline) + 1$ // The AIC of the previous best model

while $(AIC_{Current} < AIC_{Previous}\ AND\ \mathcal{C}_{\mathcal{V}} \neq \emptyset\ )$ **do**

$\mathcal{M}_{candidate}$:= `Best_Explanatory_Variable`($var = \mathcal{C}_{\mathcal{V}}$, $data = data$, $baseline = \mathcal{M}_{best}$)

$AIC_{Previous}$=$AIC_{Current}$

if $AIC(\mathcal{M}_{candidate}) < AIC_{Previous}$ **then**

$\mathcal{M}_{best} = \mathcal{M}_{candidate}$

$AIC_{Current}$= $AIC(\mathcal{M}_{candidate})$

$\mathcal{C}_{\mathcal{V}} = \mathcal{C}_{\mathcal{V}} \backslash \mathcal{V}_{Best}$ // $\mathcal{V}_{Best}$ is returned from function Best_Explanatory_Variable

return $\mathcal{M}_{best}$

return $AIC_{Current}$

End Function

In Algorithm A, we set the initial baseline model as the model which only contains an intercept.

Appendix B Analysis of the models for position

The following appendices give the parameter estimates for the 33 models characterizing the shape of the three tree crowns considered in this work. For each tree there is one model for the 11 variables presented in Table 2. The models are given in Section 4; they depend on the dependent variable x, v (or c), ℓ or n defined in Table 2. The models for the variable x (position) obtained with the three trees are given in Tables 6, 7, and 8.

Level	Variables					
	Response	Model specific	Linear		Quadratic	Interaction
2	x_2	ϕ	Intercept			
		1.63	1×10^{-3}			
3	x_3	ϕ	Intercept	ℓ_2	ℓ_2^2	
		2.70	2.42	-10.64	11.83	
4	x_4	ϕ	Intercept			
		1.430	0.59			

Table 6. Parameter estimates for the models for position for the Pixie Tree data sets. Note that the level 2 model can only have an intercept.

Level	Variables					
	Response	Model specific	Linear		Quadratic	Interaction
2	x_2	ϕ	Intercept			
		4.26	-0.45			
3	x_3	ϕ	Intercept			
		5.42	-0.18			
4	x_4	ϕ	Intercept	c_3	c_3^2	
		11.34	-18.03	41.59	-24.06	

Table 7. Parameters estimates for the models for position for the Wonder Tree data sets. Note that the level 2 model can only have an intercept.

Level	Variables							
	Response	Model specific	Linear				Quadratic	Interaction
2	x_2	ϕ	Intercept					
		3.66	-0.15					
3	x_3	ϕ	Intercept		ℓ_2			
		3.44	2.13		-4.87			
4	x_4	ϕ	Intercept	c_2		n_3		
		59.83	-0.40	0.81		0.09		

Table 8. Parameter estimates for the model for position for the Tompa Tree data sets. Note that the level 2 model can only have an intercept.

Appendix C Analysis of the model for direction

The models for the variable c (direction) obtained with the three trees are given in Tables 9, 10, and 11.

Level	Variables								
	Response	Model specific	Linear					Quadratic	Interaction
2	c_2	τ	Intercept	x_2				x_2^2	
		5.36	0.67	1.30				-1.54	
3	c_3	τ	Intercept		c_2	ℓ_2			
		3.34	1.322		-1.06	1.084			
4	c_4	τ	Intercept		c_2	ℓ_2	x_4		$x_4 * \ell_2$
		8.63	8×10^{-3}		-1.31	4.03	1.78		-3.58

Table 9. Parameter estimates for the models for the direction of the branches of Pixie Tree.

Level	Variables						
	Response	Model specific	Linear			Quadratic	Interaction
2	c_2	τ	Intercept				
		18.68	0.82				
3	c_3	τ	Intercept		x_3		
		3.79	0.64		0.57		
4	c_4	τ	Intercept	ℓ_2			
		9.27	-0.11	1.22			

Table 10. Parameter estimates for the models for the direction of the branches of Wonder Tree.

Level	Variables						
	Response	Model specific	Linear			Quadratic	Interaction
2	c_2	τ	Intercept				
		13.13	0.60				
3	c_3	τ	Intercept				
		10.21	0.60				
4	c_4	τ	Intercept	x_3	c_3		
		8.82	0.93	1.19	-0.94		

Table 11. Parameter estimates for the models for the direction of the branches of Tompa Tree.

Appendix D Analysis of the model for length

The tables below provide summaries of the explanatory variables for ℓ (length) selected with the AIC selection procedure presented in Section 4.5. The parameter estimates for the three trees are given in Tables 12, 13, and 14.

Level	Variables											
	Response	Model specific		Linear						Quadratic		Interaction
2	ℓ_2	η	Intercept	x_2	c_2					x_2^2		
		3.03	-1.90	0.96	0.95					-3.14		
3	ℓ_3	η	Intercept	x_2				x_3	c_3		x_3^2	
		2.57	-1.63	-1.06				-1.85	0.41		1.17	
4	ℓ_4	η	Intercept	x_2		ℓ_2	n_2					
		2.05	-2.40	-2.17		4.86	-0.19					

Table 12. Parameters estimates for the models for the length of the branches of Pixie Tree.

Level	Variables												
	Response	Model specific		Linear							Quadratic	Interaction	
2	ℓ_2	η	Intercept	x_2	c_2						x_2^2		
		6.37	-0.28	-4.13	0.390						2.90		
3	ℓ_3	η	Intercept			n_2	x_3	c_3				$x_3 * c_3$	$x_3 * n_2$
		4.05	-3.12			0.10	2.570	1.81				-3.10	-0.14
4	ℓ_4	η	Intercept			n_2			x_4	c_4			
		4.32	-2.98			0.06			-0.47	1.39			

Table 13. Parameter estimates for the models for the length of the branches of Wonder Tree.

Level	Variables																	
	Response	Model specific	Linear										Quadratic	Interaction				
2	ℓ_2	η	Intercept	x_2	c_2													
		2.64	-0.88	-1.63	0.88													
3	ℓ_3	η	Intercept		c_2	ℓ_2	n_2	x_3					x_3^2	$x_3 * \ell_2$	$\ell_2 * n_2$			
		3.96	3.61		-2.40	-11.94	-2.73	7.49					-4.10	-10.45	5.90			
4	ℓ_4	η	Intercept	x_2	c_2			x_3	c_3	ℓ_3	n_3	x_4				$n_3 * x_2$	$c_3 * \ell_3$	$x_4 * x_2$
		1.55	-7.52	-6.24	4.59			-1.20	5.66	7.79	1.38	-5.31				-3.83	-9.34	11.25

Table 14. Parameter estimates for the models for the length of the branches of Tompa Tree.

Appendix E Analysis of the model for the number of offspring

Tables 15, 16, and 17 provides a summary of the variables selected by the AIC for the model for the number of offspring for Pixie tree, Wonder tree and Tompa tree respectively.

Level	Variables												
	Response	Linear								Quadratic	Interaction		
2	n_2	Intercept	x_2	c_2	ℓ_2					x_2^2		ℓ_2^2	
		-0.97	1.56	1.04	12.53					-3.98		-20.39	
3	n_3	Intercept	x_2		ℓ_2	n_2	x_3	c_3	ℓ_3		c_3^2	ℓ_2^2	$c_3 * \ell_2$
		7.68	-8.34		-52.05	0.13	-3.5038	0.66	10.83		-6.37	32.63	35.91

Table 15. Parameter estimates for the models for the number of offspring branches of Pixie Tree.

Level	Variables						
	Response	Linear				Quadratic	Interaction
2	n_2	Intercept	x_2	c_2			
		0.55	-9.55	3.035			
3	n_3	Intercept			ℓ_3	ℓ_3^2	
		-9.16			35.04	-25.90	

Table 16. Parameter estimates for the models for the number of offspring branches of Wonder Tree.

Level	Variables							
	Response	Linear					Quadratic	Interaction
2	n_2	Intercept	x_2	ℓ_2				
		-2.28	-2.01	8.28				
3	n_3	Intercept		ℓ_2	n_2	ℓ_3	ℓ_3^2	
		-72.42		70.40	4.23	25.98	-24.82	

Table 17. Parameter estimates for the models for the number of offspring branches of Tompa Tree.
