# Supplementary material for: Learning how a tree branches out: A statistical modeling approach
Source: PLoS One. 2022 Sep 21;17(9):e0274168. doi: 10.1371/journal.pone.0274168 (PMC9491565; doi:10.1371/journal.pone.0274168)
Supplement: Attachment — Submitted filename: Reply_Finalv2.pdf [file pone.0274168.s003.pdf]

Responses to the reviewers' comments.

We thank the referees for their comments on our manuscript. This document contains the comments followed by our responses, in italics.

Reviewer #1: To understand this paper one needs to have read Dutilleul et al. (2015). A subset of three trees are used here. CT (computerized tomography) seems to be limited to just 'miniature' trees (ca. 20 cm tall and wide). Is there any reason to believe that, ontologically, such models will apply to mature trees?

It is true that the crowns of the Picea glauca *Pixie specimen, the* Picea glauca *Cy's Wonder specimen and the* Picea abies *Tompa specimen studied here (Manuscript #PONE-D-21-24626) were CT scanned as described in Dutilleul et al. (2015). In the* Frontiers in Plant Science *METHODS article, no attempt was made to model their branching pattern except globally, using fractal dimension estimates.*

A miniature conifer may be mature, it will be of small size, as the adjective "mature" refers to full development or age, instead of size.

Please visit https://www.mcgill.ca/ecp3/gallery-ct-scanner-canon-aquilion-prime-sp *to see an example of tree CT scanning with a non-mature non-miniature specimen; this gallery of slideshow images was produced in Summer 2020, after the macro-CT scanning equipment at the Macdonald Campus of McGill University was renewed.*

To the best of our knowledge, CT scanning data are not yet available for large trees. When that will be the case, or equivalent information is obtained with a different technology, the flexible approach described for data analysis in our PLOS ONE manuscript can be used for large trees.

It is difficult to see what this work seeks to achieve apart from applying CT scanning to very small trees. Further, the new paper seems to be a rerun of the earlier search for internal patterns to branching with fractal analysis, one that now looks for statistical correlations.

We respectfully disagree with this comment/remark for the following reasons.

The title of our PLOS ONE manuscript is "Learning how a tree branches out: A statistical approach", which we have revised by inserting "modeling" in the subtitle. By contrast, the title of Dutilleul et al. (2015, FiPS) is "Crown traits of coniferous trees and their relation to shade tolerance can differ with leaf type: a biophysical demonstration using computed tomography scanning data".

As requested elsewhere (Minor point 1), we revised the first sentence of the Abstract, but we believe our abstract was and still is clear in the following parts: "This paper … shows how the CT scanning data can be leveraged by constructing an analytical representation of a tree branching structure where each branch is represented by a line segment in 3D and classified in a level of a hierarchy, starting with the trunk (level 1). Each segment, or branch, is characterized by four variables: (i) the position on its parent, (ii) its orientation, a unit vector in 3D, (iii) its length, and

(iv) the number of offspring that it bears." and continuing with "A deeper analysis, based on statistical models aiming to explain how the characteristics of a branch are associated with those of its parents, is presented."

Above, "analytical representation" and "hierarchy" are keywords.

Simply put, we first present an original way to summarize a huge CT scanning dataset into a manageable spreadsheet thanks to the analytical representation of a tree branching pattern, and then propose statistical models/methods/procedures to analyze the relationships between structural characteristics of the tree branching pattern at and over several levels of a hierarchy.

A main concern is what is meant by a 'sample' in this paper. From my reading, the whole tree is scanned to obtain a complete skeletal structure (e.g. Fig. 1a). This not a sample in the statistical sense. In other words, the data analysis does not use repeatedly-taken random samples of branches (and sub-branches) but all the information about each tree is taken together.

In our original submission, "sample" appeared two times in the first paragraph of Section 2, the first time in a very general sense, and 5 times in the first paragraph of Section 5 (Results and discussion), in "sample mean(s)", "sample standard deviations" and "sample size(s)". It is true that we performed no sampling, since we analyzed all the branches for each tree at levels 2, 3 and 4.

Following the Reviewer's comment and to be consistent with our own terminology elsewhere in the manuscript (e.g., in Table 3), "sample" has ben replaced by "specimen" (already used in the manuscript) in Section 2, and was simply taken out of Section 5.

Since statistical distributions are applied to model the error terms (as part of the different regression fitting), using all data per tree means surely that the individual (derived) values are not independent (see Table 1). They are very highly likely to be spatially autocorrelated. Unless, I have overlooked something, or there is a part missing to the paper, the application of regression and statistical inference cannot be valid. I could find no discussion by the authors of 'sampling', 'independence' or 'randomization'.

The exact meaning of "independence" in this Comment-Response exchange is important. In regression analysis, the response variable or variable to explain (on the left side of the model equation) is sometimes said to be "dependent", while the explanatory variables (on the right side) are called "independent". Since the Reviewer mentions spatial autocorrelation, we believe the absence of correlation is the questioned form of "independence" here. The Reviewer is correct, there is no sampling or randomization in our statistical analyses because we work with all the branches at levels 2, 3 and 4 of the hierarchy for each tree (see our response to a comment above) and random errors represent the unexplained variation.

The assumption justifying our model fitting is one of "conditional independence". For example, consider the model for cosine c_3 of the level 3 branches. The assumption is that the cosines c_{3i} of level 3 branches are conditionally independent, given their parent characteristics (x_{2i}, $c_{2i,}$ l_{2i}, n_{2i}) and their position x_{3i} on their respective parent branch, as is now discussed in the first paragraph of Section 4.5.

I[PD1]*f the conditional independence assumption was violated, the estimators of model parameters would still be unbiased; in general, a misspecification of the correlation structure impacts the estimated variance of estimators and the number of degrees of freedom associated with their statistical distributions. The goal of our analyses, however, is "learning" how trees branch out by description and modeling, i.e., not testing the significance of estimated coefficients. Accordingly, we use the information criterion AIC for model selection; see Appendix A. The use of the hierarchical model presented in Section 4, with the conditional independence assumption, is suitable for such an analysis. So doing, we searched and found relationships between structural variables measured on three trees, which were chosen for the differences in complexity of their branching patterns.*

On line 225, 'sample means' and 'sample standard deviations' are referred to.

As mentioned in another Comment-Response exchange, the word "sample" has been taken away from Section 5 as part of our revision.

Examining the data frames in the DMRSuppMat.zip files it would indeed appear that all data are involved after the line-segment conversion. On line 181 it is said that there are 'eleven underlying random variables', which left me more confused. Furthermore, how can it be justified that a conditional variable becomes an explanatory one?

A dependent variable may become explanatory in a multivariate analysis. Below, we give two examples, one theoretical and one applied.

In the 4-variate case, the probability density function of the random vector (x, y, z, w) can be decomposed as the product $f(x)\, f(y|x)\, f(z|x, y)\, f(w|x, y, z)$, the vertical bar indicating conditionality. Please note that the variable y is on the left of the bar in $f(y|x)$, i.e., y is dependent on x, but on the right of the bar in $f(z|x, y)$, i.e., x and y explain z.

An example in forestry is provided by Fang et al. (2001), who model the dominant height (HD), the basal area (BA) and the volume per hectare (V) in a number of plots. In their path analysis, Fang et al. order the variables as (i) HD, (ii) BA, and (iii) V. They construct three regression models. In the first one, HD is expressed in terms of some explanatory variables. In the model for BA, the explanatory variables are those for HD, plus HD itself. Finally, both HD and BA are among the explanatory variables for V. Reference: Fang, Z., Bailey, R. L., and Shiver, B. D. "A multivariate simultaneous prediction system for stand growth and yield with fixed and random effects." Forest Science 47.4 (2001): 550-562.

Much more explanation of the rational is needed. Important issues concerning causality are involved and need addressing.

We re-read our texts and believe the rationale was/is there in sufficient quantity. Simply, so to speak, it is possible (based on the Comment-Response exchanges above) that the Reviewer was thinking about a different rationale than ours. Without repeating ourselves, we would like to add here that the statistical analysis presented in the paper targets the links between geometrical characteristics of a tree branching pattern, as measured by constructing its analytical representation from CT scanning data/images. The relationships that are identified

are not interpreted as being causal. Instead, we showcase (one of our objectives) what can be done with the new type of datasets to study tree branching structure.

The main outcome of this analysis is given on lines 305-313: that level 2 branch variables are the most important to tree form. But this will not be a surprise to many plant scientists given that major 1-D segments are being distributed (placed) within a 3-D volume (or, when more constrained, onto a 2-D plane).

Thank you very much for this comment. It is good, indeed, that the statistical analysis of the new type of datasets used in our work gives results that are in agreement with known results in the plant sciences.

And that level 4 branch variables depend upon the level 2 ones, but not on those of level 3, is not really "intriguing", at least to me, rather it is counterintuitive to what we know of the physiology and development of growing trees. Surely this aspect needs to be unravelled and fully investigated before publishing?

Yes, there is the tree physiology and the spatial distribution/space occupancy of branches at levels 2, 3 and 4; the two aspects are related but can be distinguished. We checked the results of our statistical analyses; they are OK. In a sense, this finding agrees with your previous comment, i.e., that "level 2 branch variables are the most important to tree form". They are so important to tree form that they are kept as explanatory variables in the models for the characteristics of the level 3 and level 4 branches. We revised the last paragraph of Section 5 (Results and discussion), to make the interpretation of this finding clearer.

Perhaps this 'odd' result occurs because of a mix of positive and negative correlations between variables which have a spatial auto-correlative component?

In many multivariate data analyses, some of the variables are positively correlated, or negatively correlated, or uncorrelated, and ours is not different; see Table 4. As mentioned in a Comment-Response exchange above, we do not think that models that do not rely on a conditional independence assumption would give different results. For some of the components, more sophisticated models are simply not available. For example, we do not know of a generalized beta regression model for the position variable x and a small circle model for the cosines between parent and offspring directions in that case.

In conclusion, reading this paper left me dissatisfied and sceptical about both the method and the results. No particular idea or hypothesis was being properly tested. How does such an analysis help advance our understanding of tree architecture and growth? It is more a CT technical applications report.

We respectfully disagree with this concluding remark, but can understand it because it was made on our original manuscript and before we give the explanations in the Comments-Responses exchanges above. In summary, our work is biostatistical in nature; it proposes a new framework (data and methods) for exploring tree branching structure. The goal of this paper is twofold. First, we propose "the analytical representation of a tree" as a means to summarize key characteristics of a tree branching pattern as captured by CT scanning. Second, we present

statistical models to analyze the relationships among these characteristics and apply them to three trees with branching patterns featuring various levels of structural complexity.

Minor points:
1. The Abstract has several language errors which lead to misunderstandings. 'CT' needs to be defined. The first sentence is not an appropriate way to begin.

The Abstract was revised and, hopefully, improved. In particular, the first sentence was rewritten and a number of language errors (e.g., "associated to") were corrected.

2. The Introduction on page 2 has several syntax errors. On line 12, is 'diverted' the right word? And, likewise on line 26, is 'privilege'? In some sentences there are words missing.

"diverted" seems OK. According to Merriam-Webster, it means "turned from one course or use to another".

"to privilege" is a verb that means "to accord a higher value or superior position to". It fits in the sentence.

We worked on the Introduction, at the places mentioned above and others.

3. Figs 1-3 could be easily combined with a common legend.

Such a figure would be very large, though. Maybe this can be dealt with at the final stage of copy-editing of our paper?

4. By 'beta' distribution I assume (checking the equation in other texts) that 'beta-binomial' is meant. It is not explained why this distribution was used to model position x. The beta-binomial is often employed to cater for over-dispersion. Is that the case here? I am unsure that it is the right error distribution and more care is needed to justify it.

We use the beta distribution (see the probability density function in the first equation of Subsection 4.1) to model position x. This is a continuous variable whose support is the interval (0, 1). The beta-binomial distribution is used for count data, that is, for a variable whose support is the set of integers {0, 1, 2, … }. The 4 models we use for the 4 characteristics (position, orientation, length, and number of offspring) are generalized linear models applied to the "mean" of an offspring characteristic related to the characteristics of ancestors.

5. The conclusion has at least seven spelling errors.

We worked on improving the Conclusion (linguistic and scientific aspects), by addressing this comment and incorporating comments received from another Reviewer.

6. Appendices A-E could readily go into the Supplementary Materials files.

We made no change for the time being. Maybe this can be dealt with at the final stage of copy-editing of our paper?

Reviewer #2: The authors used modeling and statistical techniques to investigate the branching pattern of trees. The manuscript and data are well organized and presented. This is an interesting approach to analyze trees structure which showed that their branching patterns have various levels of complexity.

Thank you very much for the positive assessment!

Reviewer #3: In this work, branching pattern of trees has been investigated using a statistical modeling technique. The statistical model was fitted to data derived from CT scans of three tree types. I'd recommend accepting the manuscript after the following minor changes.

Thank you very much for the positive assessment!

-Please discuss how the presented method and derived variables/coefficients for three modeled trees could be expanded to other tree types?

The following text has been added to the end of the first paragraph of the conclusion: "The 4 geometric characteristics of the analytical representation defined in Section 3 have the same range, regardless the size and species of the tree. The position x is in the interval (0, 1), v is a 3D unit vector and the length l (relative to that of the trunk) is a positive variable, while the number n of offspring is a non-negative integer. Thus, the models proposed in Subsection 4.1 are widely applicable and could be extended to many other trees."

That said, more work on CT scanned trees is needed to ascertain whether the descriptive statistics of Table 3 and the coefficients of fitted models in Appendices B to E apply to wide classes of trees.

-Please discuss how the results will be changed if the modeling is repeated with more trees of the same type. Are the results reported in this work conclusive for three tree types that were investigated, or the model needs to be fitted to more CT data of the same tree type in the future?

With one tree per species, we cannot state that we have conclusive results for three species. The next step for this would be to consider several trees of the same species in order to assess the within-species variability in the application of our models. This is now stated in the third paragraph of the conclusion.

-Please discuss the possibility of developing a universal model that accounts for all parameters including tree age and environmental factors.

This is ambitious, but since we "live with our dreams", we included a short discussion of this possibility in the fourth paragraph of the revised Conclusion.
