# Supplementary material for: Learning how a tree branches out: A statistical modeling approach
Source: PLoS One. 2022 Sep 21;17(9):e0274168. doi: 10.1371/journal.pone.0274168 (PMC9491565; doi:10.1371/journal.pone.0274168)
Supplement: Attachment — Submitted filename: Response_to_reviewers_R2.pdf [file pone.0274168.s004.pdf]

Response to the comments of the academic editor on “PONE-D-21-24626R1 Learning how a tree branches out: A statistical modeling approach”

more in-depth quantitative analysis of branching pattern.

The model that are fitted are relatively complex so in this revision we tried to explain them better. Here are the steps taken to address this comment:

- *Section 4.5 have been modified to emphasized the dependency of the models on parent characteristics. The term ”conditional independence” was confusing. It has been removed*
- *The Supplementary information now has a file with the R-code needed to replicate the findings in Table 12 of the paper.*
- *All the suggestions made by the referee to improve the content of the paper have been implemented.*

Response to the comments of A. Flores-Palacios on “PONE-D-21-24626R1 Learning how a tree branches out: A statistical modeling approach”

Dear Prof. Flores-Palacios,

We sincerely thank you for a positive and constructive evaluation of our paper. Below are our responses to the comments in your PLOS-ONE review report.

1-Your answer to the question “Have the authors made all data underlying the findings in their manuscript fully available?” is NO.

We respectfully disagree with this answer. The three spreadsheets that are at the basis of our data analysis because they contain the analytical representations of the branching patterns of the three trees used as examples in the paper are available in the Supplementary Material; see Supporting information. These files were prepared using the techniques described in Section 3. As shown in Table 1, each spreadsheet has 27 columns. The number of rows of a spreadsheet is equal to the number of terminal branches of the tree (this is discussed before Table 1); it generally varies from tree to tree.

Note: Following the first part of Comment c, the updated Supplementary Material also contains an R script, “basic_example.R”, which fits the length models for Pixie Tree using the dataset “CTobsn.txt", with the analytical representation of Pixie Tree.

2-Review Comments to the Author

Reviewer #4: In the MS "Learning how a tree branches out: A statistical modeling approach," the authors analyzed the data from three small trees. I do believe that this effort could be beneficial, but:

a.- Trees are the dominant life form in all the forests, and understanding their architecture is not only crucial for modeling their photosynthetic capacity. Trees are the minimal habitat for canopy organisms such as epiphytes and insects. In this regard, several authors have tried to test how trees' complexity affects epiphyte diversity, see:
Ruiz-Cordova, J. P., V. H. Toledo-Hernández and A. Flores-Palacios. 2014. The effect of substrate abundance in the vertical stratification of Bromeliad epiphytes in a tropical dry forest (Mexico). Flora 209: 375–384.
Flores-Palacios, A. and J. G. García-Franco. 2006. The relationship between tree size and epiphyte richness: testing four different hypotheses. Journal of Biogeography 33: 323–330.
Victoriano-Romero, E., S. Valencia-Díaz, V. H. Toledo-Hernández and A. Flores-Palacios. 2017. Dispersal limitation of Tillandsia species correlates with rain and host structure in a central Mexican tropical dry forest. PLoS ONE 12(2): e0171614.
At least one of these papers measures most of the tree structures.

Thank you very much for this comment and the references. If measurable, variables of the analytical representation of a tree branching pattern discussed in Section 3 could be used as explanatory variables in analyses like those in the references mentioned above. For instance, branch orientation (say, relative to the Earth’s surface) could be an important predictor for the models proposed in your 2014 and 2017 papers, that is, branches, or the “woody structure”,

orthogonal to the dominant wind direction could have higher epiphyte abundance. These two articles are cited in the introduction and the conclusion in our newly revised manuscript.

b.- All trees have a hierarchy of structures; for example, the first trunk division is not independent of the trunk, and so on. So the different structures and their properties must be modeled considering that they are not independent.

That is essentially what we do. The models proposed in Section 4 account for a dependence between structures, as explained at the beginning of Sub-section 4.5: "The models proposed in this section decompose the joint density for the eleven underlying random variables in terms of the marginal density for x_2 times the conditional density of v_2 given x_2 times the conditional density of ℓ_2 given x_2 and v_2 and so on." The use of conditionals makes the link from one structure to the next in our modeling. The only independence assumption that is made is between the characteristics of two branches at the same level in the hierarchy. For example, given all their parent characteristics, the lengths of any two branches at level 3, say, are statistically independent.

Dependence can be found in the models that are fitted. Consider, for instance, the length of level-3 branches (ℓ_3) for Pixie Tree. The fitted model given in Table 12 expresses the scale parameter of the Weibull distribution for ℓ_3 as: $-1.61 -1.06\ x_2 -1.85\ x_3 +0.41\ c_3 -1.17\ x_3^2$. The coefficient -1.06 for x_2 means that level-3 branches whose parents are at the end of the trunk (with large x_2) are shorter than branches on parents that are closer to the ground (with small x_2). The other coefficients are interpreted in a similar way.

c.- The model description is complex (I understand this), making the replicability of the modeling will be limited. Can you provide a simple r-library for the use of your model?

The Supplementary Material now includes a link to an R file with code performing the analysis for the branch lengths of Pixie Tree. It reproduces the results reported in Table 12.

Can you give a single number that will describe branching patterns or tree complexity? If yes, how can this number be used for comparing tree species?

We assume that Reviewer #4 means "describe a branching pattern" (singular). When planning our study, we wanted to consider trees with varying structural complexity of the branching pattern, and we used the fractal dimension (FD) as 'universal measure of complexity' for this purpose. After a brief mention of fractal analysis in the Introduction, we further discussed the matter on Page 17: "To begin with, these three trees were chosen from the 15 conifers studied by Dutilleul et al. (2015) because the structural complexity of their branching pattern ranged from high (Pixie Tree) to low (Tompa Tree) with Wonder Tree in-between. This was measured with fractal dimension estimates (FD) in Dutilleul et al. (2015)."

Will your model help test ecological hypotheses like those in previous articles?

The analytical representation of the branching pattern of a tree contains information that could be useful to test ecological hypotheses; see our response to Comment a. The models presented in Section 4 could be used in subsequent analyses to relate ecological findings and tree structure.

Join Figures 1, 2, and 3.

This has been done; see the new Figure 1 with six panels (a) – (f).
